# P-589. Cholesterol Control in Virologically Suppressed People with *HIV-1* Switching to Tenofovir DF-containing, Ainuovirine-Based Compared to Tenofovir Alafenamide-Containing, Boosted Elvitegravir-Based Antiretroviral Regimen: Hypercholesterolemic Subgroup Analyses of the SPRINT Trial, a Randomized, Active-Controlled Phase 3 Study

**DOI:** 10.1093/ofid/ofae631.787

**Published:** 2025-01-29

**Authors:** Fujie Zhang, Hao Wu, ping ma, Qingxia Zhao, hongxia wei, Hongzhou Lu, Hui Wang, Shenghua He, Zhu Chen, yaokai Chen, Ming wang, Weiping Cai, Hong Qin

**Affiliations:** Beijing Ditan Hospital, Beijing, Beijing, China; Beijing Youan Hospital Affiliated to Capital Medical University, Beijing, Beijing, China; Nankai University Second People's Hospital, School of Medicine, Nankai University, tianjin, Tianjin, China; Zhengzhou Municipal Sixth People’s Hospital and Infectious Disease Hospital of Henan Province, Zhengzhou, Henan, China; The Second Hospital of Nanjing, Nanjing University of Chinese Medicine, Nanjing, Jiangsu, China; Shenzhen Municipal Third Hospital, Shenzhen, Guangdong, China; Shenzhen Municipal Third Hospital, Shenzhen, Guangdong, China; Chengdu Municipal Public Health Clinical Center, Chengdu, Sichuan, China; Chengdu Municipal Public Health Clinical Center, Chengdu, Sichuan, China; Chongqing Public Health Medical Center, chongqing, Chongqing, China; The First Hospital of Changsha, Changsha, Hunan, China; Guangzhou Municipal Eighth People’s Hospital Affiliated to Guangzhou Medical University, Guangzhou, Guangdong, China; Jiangsu Aidea Pharmaceutical Co., Ltd, Yangzhou, Jiangsu, China (People's Republic)

## Abstract

**Background:**

In the SPRINT trial, virologically suppressed people with HIV-1 (PWHs) switched to tenofovir DF (TDF) containing, ainuovirine (ANV, ACC008) based or tenofovir alafenamide (TAF) containing, boosted elvitegravir (EVG/c, comparator) based antiretroviral (ARV) regimen from efavirenz-based regimen.

**Figure 1.**
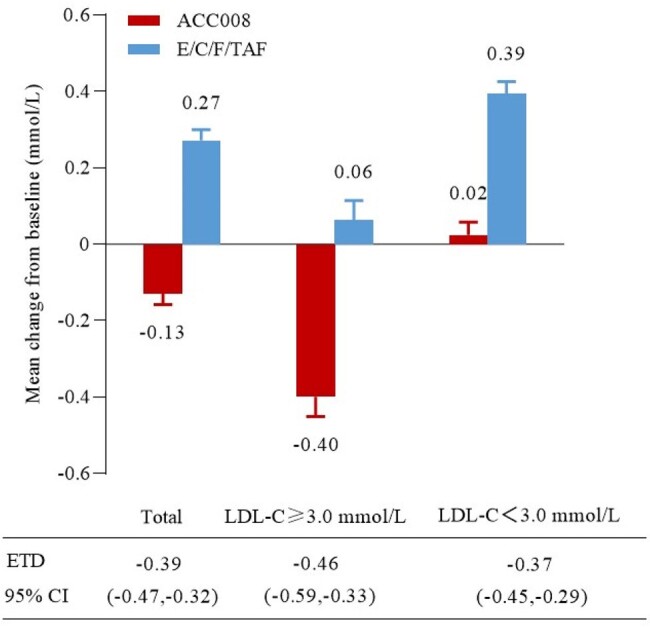
CFB in LDL-C at week 48 by baseline level. ACC008, ainuovirine/ lamivudine/ tenofovir disoproxil; CFB, change from baseline; E/C/F/TAF, elvitegravir/ cobicistat/ emtricitabine/ tenofovir alafenamide; LDL-C, low density lipoprotein cholesterol.

**Methods:**

Baseline hypercholesterolemia was defined as serum LDL-C at 3.0 mmol/L or above; acceptable lipidemic control was defined as post-treatment serum LDL-C below 3.0 mmol/L, and good lipidemic control as post-treatment serum LDL-C below 2.6 mmol/L as recommended by the European AIDS Clinical Society. Changes from baseline (CFBs) in LDL-C (least square mean) at week 48 were compared using the mixed effects models for repeated measures with treatment as fixed factor and baseline LDL-C measurement as a covariate, all nested within visits. The proportions of PWHs with good/acceptable lipidemic control were analyzed using the logistic regression method with baseline serum LDL-C adjusted at week 48.

**Figure 2.**
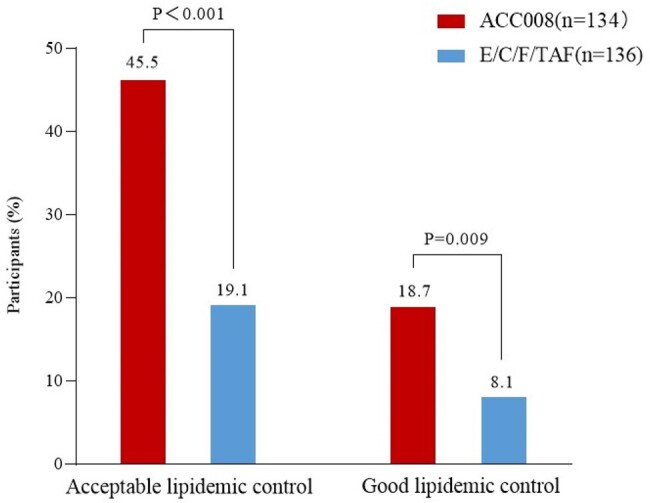
Proportions of participants with acceptable and good LDL-C control at week 48 ACC008, ainuovirine/ lamivudine/ tenofovir disoproxil; E/C/F/TAF, elvitegravir/ cobicistat/ emtricitabine/ tenofovir alafenamide; LDL-C, low density lipoprotein cholesterol. Acceptable lipidemic control, LDL-C＜3.0 mmol/L; Good lipidemic control, LDL-C＜2.6 moml/L.

**Results:**

The proportions of PWHs with hypercholesterolemia were comparable between the two arms (35.2% [134/381] vs. 35.7% [136/381]) with a similar mean value of 3.49 ± 0.42 vs. 3.52 ± 0.40 mmol/L at baseline. CFB in LDL-C showed a significantly greater decline in ACC008 arm compared to that in comparator arm at week 48 (-0.40 vs. 0.06 mmol/L, estimated treatment difference [95%CI], -0.46 mmol/L [-0.59, -0.33], p< 0.001). The proportions of PWHs with acceptable/good lipidemic control were significantly greater in ACC008 arm than those in comparator arm (acceptable, 45.5% [61/134] vs. 19.1% [26/136], 0.264 [0.154, 0.366], p< 0.001; good, 18.7% [25/134] vs. 8.1% [11/136], 0.106 [0.024, 0.188], p< 0.001) at week 48.

**Conclusion:**

Hypercholesterolemia was prevalent in virologically suppressed PWHs on efavirenz-based ARV regimen. Switch to TDF-containing, ANV-based regimen resulted in greater LDL-C decline and better lipidemic control compared to that to TAF-containing, EVG/c-based regimen.

**Disclosures:**

**Hong Qin, MD, PhD**, Jiangsu Aidea Pharmaceutical Co., Ltd: Honoraria

